# Influence of Solvent Lipid Extraction on Tissue‐Specific Carbon Isotopes

**DOI:** 10.1002/ece3.73829

**Published:** 2026-06-12

**Authors:** J. Groß, B. Fry, J. Eggebo, S. Bengtson Nash

**Affiliations:** ^1^ Southern Ocean Persistent Organic Pollutants Program, Centre for Planetary Health and Food Security Griffith University Nathan Queensland Australia; ^2^ Alfred‐Wegner‐Institute Helmholtz‐Centre for Polar and Marine Research Bremerhaven Germany; ^3^ Helmholtz Institute for Functional Marine Biodiversity at the University of Oldenburg (HIFMB) Oldenburg Germany; ^4^ Australian Rivers Institute Griffith University Nathan Queensland Australia

**Keywords:** blubber, bovine, bulk stable isotopes, dietary evaluation, humpback whales, shark, solvent extraction, Southern Ocean

## Abstract

Cetacean blubber and skin have a high lipid content, which is depleted in ^13^C and ^15^N compared to proteins. As such, it is necessary to normalise samples for lipids prior to bulk stable isotope analysis. This is often achieved via solvent lipid removal. The direct effects of solvents used in lipid extractions on δ^15^N of animal tissue is a widely discussed topic in the fields of stable isotope and cetacean trophic ecology. The effects of such solvents on δ^13^C on the other hand, beyond the desired effect of lipid removal, have not yet been studied, although outcomes may be as unpredictable as those found for δ^15^N. Here, we evaluate the effects of solvent lipid extraction on four pure proteins, as well as humpback whale blubber tissue. Lipids were extracted multiple times prior to stable isotope analysis, which also served to demonstrate the efficiency of each lipid extraction cycle. Our results showed that the solvents used, dichloromethane and methanol, did not have a significant effect on δ^13^C and δ^15^N of pure protein as values largely remained within 0.5‰ of unextracted samples, which is equivalent to the expected analytical error. Humpback whale blubber δ^13^C, on the other hand, increased significantly with each lipid extraction cycle, and C:N ratios were only equivalent to those of pure protein, 2.87, after three lipid extractions. These results highlight that solvent lipid extraction has no effect on δ^13^C beyond the desired effect of lipid removal but that gentle lipid extraction protocols are inefficient and that multiple lipid extraction cycles are required to achieve robust isotopic measures.

## Graphical abstract

To assess the impact of solvents on δ^13^C, we used a standard lipid extraction protocol on four different pure proteins and humpback whale blubber, a very lipid‐rich tissue. The results showed that the solvents did not have a significant effect on δ^13^C and δ^15^N of pure proteins as values largely remained within 0.5‰ of unextracted samples, which is equivalent to the expected analytical error.

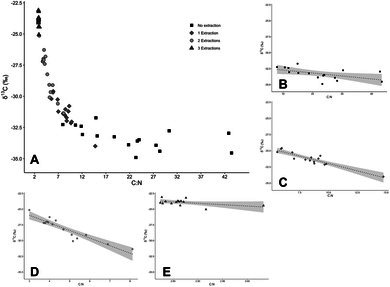

## 1 Introduction

Carbon (δ^13^C) and nitrogen (δ^15^N) stable isotope ratios are a commonly used tool to study the diet and trophic dynamics of organisms (Vandermerwe and Vogel 1978). δ^15^ are indicative of the trophic position of an animal as they increase from one trophic level to the next (Minagawa and Wada 1984). The lighter ^14^N is preferentially excreted, which results in ^15^N enrichment of 2‰–4‰ in consumers, compared to their diet (Deniro and Epstein 1981). δ^13^C indicate the isotopic composition of a food web's primary production because carbon does not fractionate significantly along the food chain (0.5‰), preserving δ^13^C of the photosynthetic pathway (DeNiro and Epstein 1978; McCutchan et al. 2003; Post 2002). However, δ^13^C are known to vary with the lipid content of a tissue because lipids are depleted in ^13^C relative to proteins or carbohydrates (DeNiro and Epstein 1978). As lipid content tends to be highly variable between different tissue types and species, as well as within a species (McConnaughey and McRoy 1979), trophic ecology interpretations may be biased (Kiljunen et al. 2006). To address this issue, there is a consensus in the field that lipid content must be accounted for (Clark et al. 2019; Post et al. 2007). Lipids can either be extracted using solvents prior to stable isotope analysis or, alternatively, δ^13^C can be standardised after stable isotope analysis using mathematical models (Fry 2002; Groß et al. 2021; Lerner and Hunt 2022; McConnaughey and McRoy 1979).

During lipid extraction, hexane‐isopropanol or methanol‐chloroform solvent mixtures are commonly used to remove lipids or reduce concentrations to a low and uniform level (Bligh and Dyer 1959; Folch et al. 1957; Pinnegar and Polunin 1999). Lipid extraction relies on the assumption that the solvents used for extraction do not alter δ^15^N significantly due to the absence of nitrogenous components in the solvents. However, several studies on a variety of freshwater and marine invertebrates and vertebrates have documented an enrichment in δ^15^N post lipid extraction (Groß et al. 2021; Mintenbeck et al. 2008; Murray et al. 2006; Ouellet et al. 2024; Riverón et al. 2022; Sotiropoulos et al. 2004; Sweeting et al. 2006). Other studies have documented negative or no effects of lipid extraction on δ^15^N (Bodin et al. 2007; Logan et al. 2008; Ryan et al. 2012). The observed fractionation of δ^15^N is hypothesised to result from solvents potentially solubilising amino acids (Pinnegar and Polunin 1999; Sotiropoulos et al. 2004).

Prior research investigating the effects of lipid extraction on δ^15^N of cetacean species has found varying results, even within the same species and tissue. For example, Ryan et al. (2012) found no change in northern hemisphere humpback whale ( *Megaptera novaeangliae* ) skin δ^15^N following lipid extraction using a Soxhlet reflux extraction with hot *n*‐hexane and acetone while Groß et al. (2021) found a significant increase in southern hemisphere humpback whale skin δ^15^N following a modified Bligh and Dyer (1959) methanol‐dichloromethane‐water lipid extraction at room temperature. Lesage et al. (2010) found a significant decrease in δ^15^N of Balaenopteridae skin following lipid extraction using a solvent mixture of methanol and chloroform at 4°C. Due to these contrasting changes in δ^15^N post lipid extraction, the question arose whether the solvents used in lipid extractions also alter δ^13^C in an unpredictable manner in addition to reducing or removing lipids. Addressing the uncertainty that accompanies this question is especially prudent in mysticeti research as the true diet of these cetaceans is difficult to gauge due to the impossibility of conducting controlled feeding experiments on captive animals. The diet of free‐roaming mysticetes can only be estimated via direct (Lammers et al. 2024; Owen et al. 2017; Stockin and Burgess 2005) or remote‐controlled observations (Torres et al. 2018, 2020), fatty acids (Groß et al. 2020; Waugh et al. 2012) and stable isotope analyses (Eisenmann et al. 2016; Haro et al. 2021; Johnson and Davoren 2021; Riekenberg et al. 2021; Ryan et al. 2014), other ecological tracers (Eisenmann et al. 2017), or post‐mortem stomach analysis (Santos et al. 2001). Therefore, it is pertinent to improve the accuracy of available methods by testing the assumptions that these methods are based on.

In addition to evaluating the validity of methodological assumptions, ongoing research is needed to untangle which tissues reflect recent and/or historical feeding by free‐roaming mysticetes. Analysis of whole body or internal organs such as muscle or liver is often problematic, and consequently, there is a bias towards analysis of superficial tissues. Groß et al. (2021) recommended the use of skin tissue for dietary assessments of southern hemisphere humpback whales as skin tissue is widely accessible, uniform across the body, and lipid‐extracted C:N ratios fall within the range of pure tissues such as collagen. Nevertheless, the authors acknowledged that the intrinsic influence of diet may be more readily apparent in blubber tissue (Groß et al. 2021) because it mainly consists of lipids in the form of fatty acids, which are deposited into blubber tissue with little modification or in a predictable manner (Budge et al. 2006). Additionally, there is also a difference in isotopic turnover rate between blubber and skin (Busquets‐Vass et al. 2017; Teixeira et al. 2022). The authors favour the use of skin tissue over blubber tissue because the average blubber C:N ratio (3.9 ± 0.86; Groß et al. 2021) was higher than those expected for pure tissues such as collagen or keratin (2.8 and 3.0, respectively; Newsome et al. 2010). These higher C:N ratios suggest that lipids were not fully extracted from the blubber (Newsome et al. 2010), which means that the reported δ^13^C were lower than the actual δ^13^C, leading to interpretation biases dependent on lipid extraction efficiencies. Repeated extraction may therefore result in more accurate dietary assessments via cetacean blubber tissues.

Here, we test whether the solvent sequences used in the modified Bligh and Dyer (1959) extraction method alter the δ^13^C and δ^15^N of four types of pure proteins and humpback whale blubber collected from the east coast of Australia migrating population (described as E1 by the International Whaling Commission; hereafter referred to as ‘E1 humpback whales’). Testing pure proteins allows us to assess whether the extraction solvents alter δ^13^C beyond the desired effect of creating lipid‐free samples. Secondly, we determine whether a second and third round of solvent extraction of blubber samples leads to stable lipid concentration levels as indicated by uniform C:N ratios and C:N in the range of pure tissues such as collagen or keratin. A modified Bligh and Dyer (1959) methanol‐dichloromethane‐water extraction technique is tested for its efficiency in removing lipids from humpback whale blubber. Efficiency is assessed using C:N ratios as a proxy for total lipid content.

## 2 Materials and Methods

### 2.1 Sample Collection

In total, 15 blubber biopsy samples of free‐roaming E1 humpback whales were collected during their annual northward migration (June, July) to breeding grounds, and southward migration (September, October) to feeding grounds in 2017 (*n* = 4), 2019 (*n* = 8), and 2020 (*n* = 4). A modified 0.22 calibre rifle (Paxarms NZ) with flotation darts was used to collect the biopsies off North Stradbroke Island, southeast Queensland, Australia (approximately 27°26′ S, 153°34′ E). A biopsy was taken from the whale's dorsum, slightly posterior and ventral to the dorsal fin, as recommended by Lambertsen et al. (1994). Immediately upon collection, tissue samples were sub‐sectioned and stored on ice onboard the vessel until transferred to a −80°C freezer for long‐term storage prior to analysis. For more details, see Waugh et al. (2012). Long‐term storage at −80°C after flesh freezing samples in liquid nitrogen upon collection is the recommended method to prevent lipid degradation and hydrolysis (Couturier et al. 2020). Additionally, all samples were checked for their free fatty acid content and polyunsaturated fatty acid content, indicators for lipid hydrolysis and oxidation, respectively (Couturier et al. 2020; Groß et al. 2020). None of the blubber samples used in the analyses were degraded. The four protein laboratory standards (casein, bovine collagen, bovine serum, shark cartilage) were sourced from the *Queensland Health Forensic and Scientific Services*, Coopers Plains, Australia. All biopsy samples were collected under an animal ethics approval permit (ENV/18/18/AEC) granted by the *Griffith University Animal Ethics Committee*.

### 2.2 Lipid Extraction

Pre‐weighed (ca. 0.03 g) blubber and protein samples were solvent‐extracted overnight in closed, stationary separation funnels, at room temperature, via the routinely utilised, modified Bligh and Dyer (1959) method (Tables S1 and S2), which uses a methanol‐dichloromethane‐water (2:1:0.8 v/v/v MeOH/CH_2_Cl_2_/H_2_O) mixture. Through the addition of dichloromethane and water to the separation funnel, the aqueous and the dichloromethane phases were separated the following day, yielding a solvent ratio of 1:1:0.9 v/v/v methanol‐dichloromethane‐water. After phase separation, the total lipid content of each sample was obtained by collecting the dichloromethane phase in round‐bottom flasks, reducing it to dryness for approximately 30 min, and re‐weighing it. Before storing the extracted tissue at −18°C, it was removed from the solvent mixture and air‐dried.

### 2.3 Stable Isotope Analysis

Replicate blubber and protein samples of each type were analysed for carbon and nitrogen stable isotopes. Blubber samples and protein components were analysed with one control and three treatments. The control was analysed for stable isotopes prior to solvent extraction (hereafter referred to as ‘bulk’) and the three treatments refer to successive solvent extraction treatments (hereafter referred to as ‘lipid‐free1’, ‘lipid‐free2’, and ‘lipid‐free3’, respectively). At 58°C, all samples were oven‐dried overnight, ground to powder, and 1–2 mg of powdered sample was placed into tin capsules for δ^13^C and δ^15^N analysis. For carbon and nitrogen, the international standards used in the equation are Vienna Pee Dee Belemnite and N_2_ in air, respectively. International standards IAEA‐CH‐6 for carbon and IAEA N1 for nitrogen were used to calibrate laboratory standards (NH_4_)_2_SO_4_ and sucrose. For analysis, a Europa EA‐GSL, interfaced to a SERCON Hydra 20–20 isotope ratio mass‐spectrometer (IRMS) was used. The following formula was used to calculate stable isotope abundances in units of permil (‰):

$$\delta X=\left[\left(\frac{R_{\text{sample}}}{R_{\text{standard}}}\right)-1\right]\times 1000$$

where *X* = ^13^C or ^15^N and *R* = the respective ratio (^13^C/^12^C or ^15^N/^14^N). The analysis of replicate standards resulted in average standard deviations of 0.1‰ and 0.2‰ for δ^13^C and δ^15^N, respectively. Atomic C:N ratios were calculated and used as a proxy for lipid content (Logan et al. 2008).

### 2.4 Statistical Analysis

Analyses of stable isotope data were performed in R (version 3.5.3) and PRIMER v7 (Clarke and Gorley 2015) with PERMANOVA+ add‐on (Anderson et al. 2008; http://www.primer‐e.com). A Levene's test was used to check data for homogeneity of variance, a Shapiro–Wilk test was used to check data for normality, and a PERMDISP test was used to check for homogeneity of multivariate dispersions. The tests showed that δ^13^C and δ^15^N were not normally distributed, but sample variances were homogenous, and no difference was detected in the within‐group multivariate dispersion among groups (PERMDISP: pseudo‐*F*_2,54_ = 2.6002; *p* = 0.2769). Hence, PERMANOVAs were used to test for significant differences in blubber and protein stable isotope values between the control and the three treatments. A significance level of *α* = 0.05 was used to interpret all results. For all multivariate statistical analyses, Euclidean distance matrices were calculated. Blubber samples and protein components were analysed as bulk and lipid‐free replicates. Blubber samples collected at two different migration time points and in three different years were included in this study. Permutational multivariate analysis of variance (PERMANOVA) results showed that there was no significant difference between migrations or years (Migration: pseudo‐*F*_1,30_ = 2.3158, *p* = 0.1332; Year: pseudo‐*F*_2,30_ = 1.4124, *p* = 0.2522). Therefore, all samples were pooled in subsequent analyses. Differences between bulk (δ^13^C_B_, δ^15^N_B_) and lipid‐free values (δ^13^C_LFX_, δ^15^N_LFX_) were calculated as δ^13^C_B_ − δ^13^C_LFX_ and δ^15^N_B_ − δ^15^N_LFX_, respectively. A two‐factor PERMANOVA with the partial sum of squares (Type III) and permutation of residuals under a reduced model with 9999 permutations was used to test for differences between the control and treatments. We tested for significant differences among factor levels using *post hoc* pairwise comparisons. An instrument and laboratory error of 0.5‰ was used in this study. The typical instrument error is 0.2‰ to 0.3‰ (Jardine and Cunjak 2005). To account for laboratory errors as well, an error level of 0.5‰ was chosen for comparisons of extractions among pure protein and humpback whale blubber samples.

## 3 Results

### 3.1 Changes in Pure Protein

**FIGURE 1:**
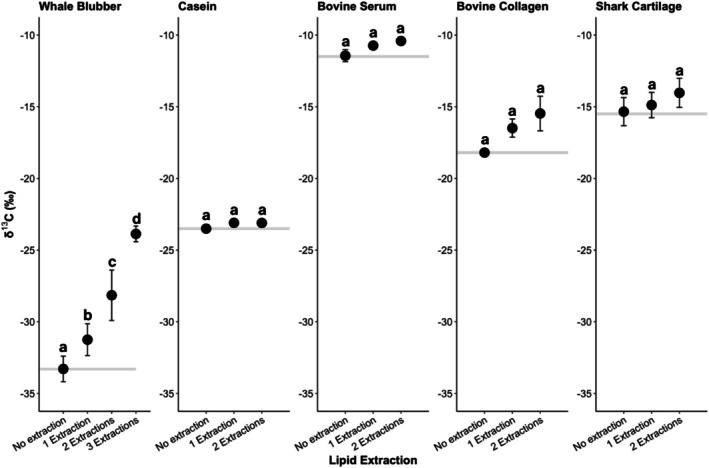
Scatterplot showing the average δ^13^C ± standard deviation of humpback whale blubber (*n* = 15), casein (*n* = 3), bovine serum (*n* = 3), bovine collagen (*n* = 3), and shark cartilage (*n* = 3) for the control (no extraction) and three treatments (extraction 1, 2, 3). The grey bar highlights the expected instrument error of 0.5‰. Where error bars are absent, the standard deviation is so small that error bars are not visible. Different lower‐case letters denote a significant difference among the different lipid extraction treatments, respectively, for humpback whale blubber and each pure protein.

**FIGURE 2:**
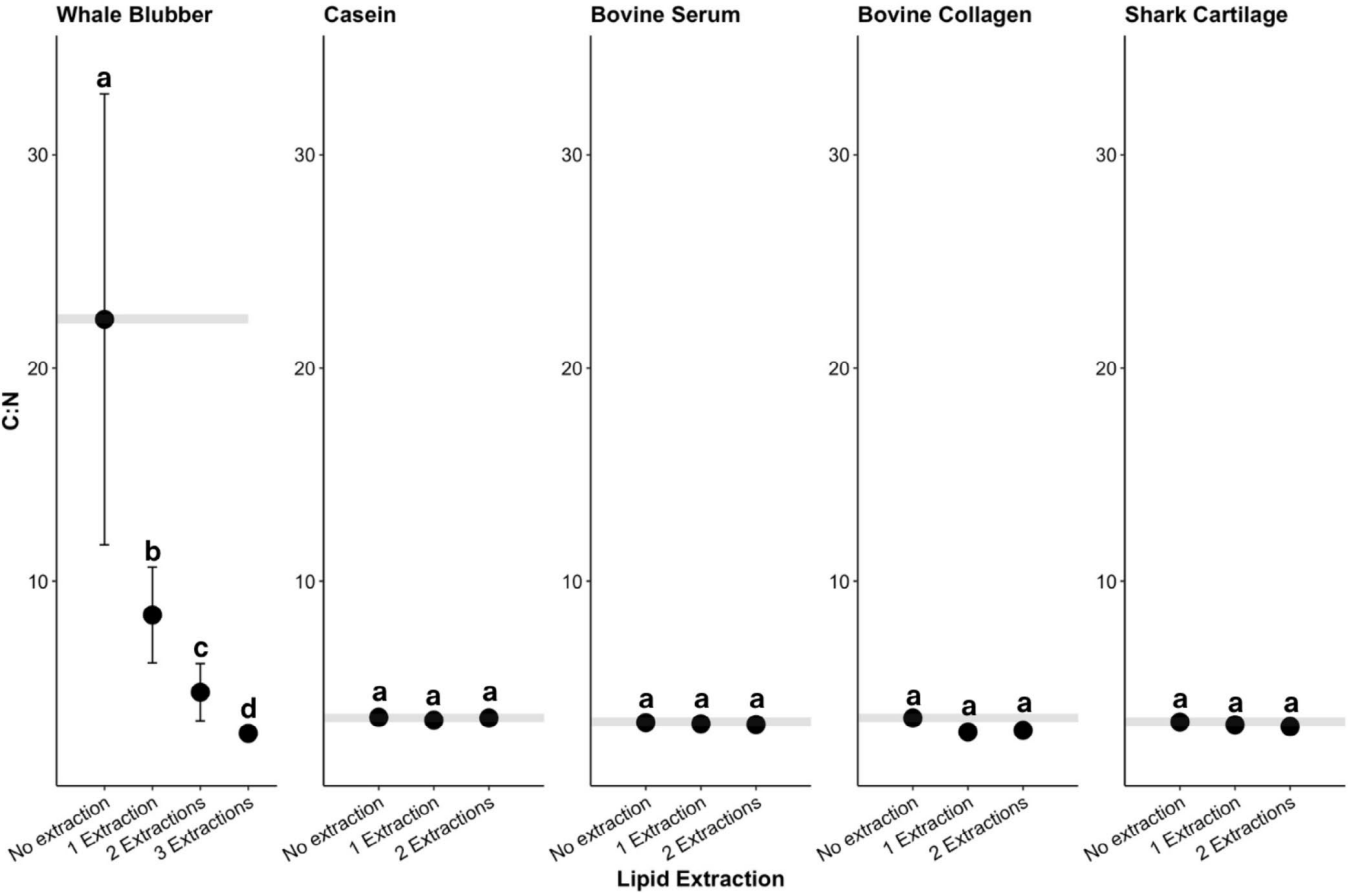
Scatterplot showing the average C:N ± standard deviation of humpback whale blubber (*n* = 15), casein (*n* = 3), bovine serum (*n* = 3), bovine collagen (*n* = 3), and shark cartilage (*n* = 3) for the control (no extraction) and three treatments (lipid extraction 1, 2, 3). The grey bar highlights the expected instrument error of 0.3. Where error bars are absent, the standard deviation is so small that error bars are not visible. Different lower‐case letters denote a significant difference among the different lipid extraction treatments, respectively, for humpback whale blubber and each pure protein.

**FIGURE 3:**
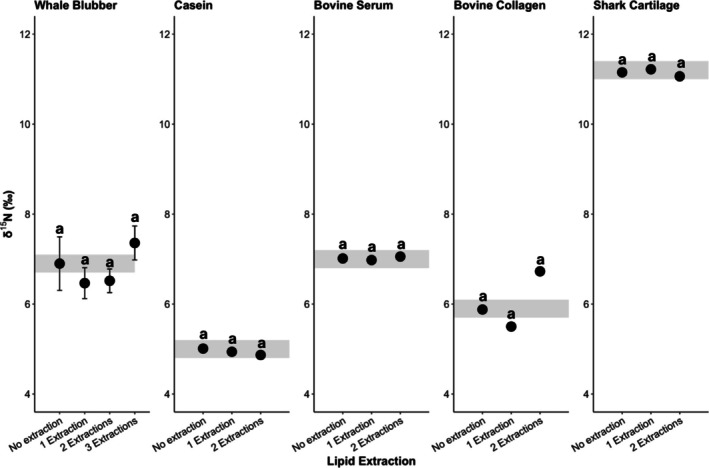
Scatterplot showing the average δ^15^N ± standard deviation of humpback whale blubber (*n* = 15), casein (*n* = 3), bovine serum (*n* = 3), bovine collagen (*n* = 3), and shark cartilage (*n* = 3) for the control (no extraction) and three treatments (extraction 1, 2, 3). The grey bar highlights the expected instrument error of 0.5‰. Where error bars are absent, the standard deviation is so small that error bars are not visible. Different lower‐case letters denote a significant difference among the different lipid extraction treatments, respectively, for humpback whale blubber and each pure protein.

Casein, bovine collagen, bovine serum, and shark cartilage δ^13^C did not vary significantly between the control and the lipid extraction treatments (Table 1). δ^13^C of casein even stayed within the range of the expected analytical instrument error of 0.5‰ after both lipid extractions, while the δ^13^C of shark cartilage only remained within the instrument error range after the first extraction (Figure 1). Bovine collagen and bovine serum δ^13^C were outside the range of the expected instrument error after the first and second lipid extraction (Figure 1). There was no significant difference in the average protein C:N ratios between the control and the treatments (Table 1). The average C:N ratio of casein, bovine serum, and shark cartilage showed no change after each lipid extraction treatment, and treatment averages remained within 0.3 of the control (Figure 2). The average C:N ratio of bovine collagen showed changes after the first and second lipid extraction (Figure 2). The average C:N ratios of both extractions of bovine collagen deviated by more than 0.3 from the control (Table 1). The average δ^15^N of all protein tissues did not vary significantly between the control and the lipid extraction treatments (pseudo‐*F*_10,37_ = 1.0629, *p* = 0.3347), which was shown by a PERMANOVA. The average δ^15^N of casein, bovine serum, and shark cartilage also stayed within the expected instrument error range after lipid extraction, but the δ^15^N of bovine collagen fell outside this range after the second lipid extraction (Figure 3).

**TABLE 1:** Average δ^13^C, δ^15^N, and C:N plus standard deviation of humpback whale blubber (*n* = 15), casein (*n* = 3), bovine serum (*n* = 3), bovine collagen (*n* = 3) and shark cartilage (*n* = 3) for the control (unextracted) and three treatments (extraction 1, 2, 3).

		Whale blubber		Casein		Bovine collagen		Bovine serum		Shark cartillage	
		Mean	SD	Mean	SD	Mean	SD	Mean	SD	Mean	SD
𝛿^13^C	Unextracted	−33.29	0.89	−23.50	0.21	−18.19	0.87	−11.44	0.42	−15.34	0.98
	1 Extraction	−31.25	1.11	−23.09	0.06	−16.49	0.64	−10.73	0.25	−14.88	0.89
	2 Extractions	−28.15	1.76	−23.10	0.06	−15.47	1.21	−10.41	0.11	−14.03	1.02
	3 Extractions	−23.87	0.55								
𝛿^15^N	Unextracted	6.89	0.59	5.01	0.16	5.88	0.29	7.01	0.23	11.15	0.54
	1 Extraction	6.46	0.34	4.94	0.29	5.50	0.35	6.98	0.39	11.21	0.28
	2 Extractions	6.52	0.26	4.87	0.49	6.73	2.59	7.06	0.53	11.06	0.29
	3 Extractions	7.36	0.38								
C:N	Unextracted	22.28	10.57	3.61	0.18	3.58	0.11	3.37	0.13	3.39	0.21
	1 Extraction	8.40	2.25	3.49	0.06	2.93	0.08	3.28	0.07	3.27	0.11
	2 Extractions	4.79	1.35	3.58	0.09	3.01	0.35	3.32	0.04	3.19	0.05
	3 Extractions	2.87	0.05								

### 3.2 Changes in Humpback Whale Blubber

**FIGURE 4:**
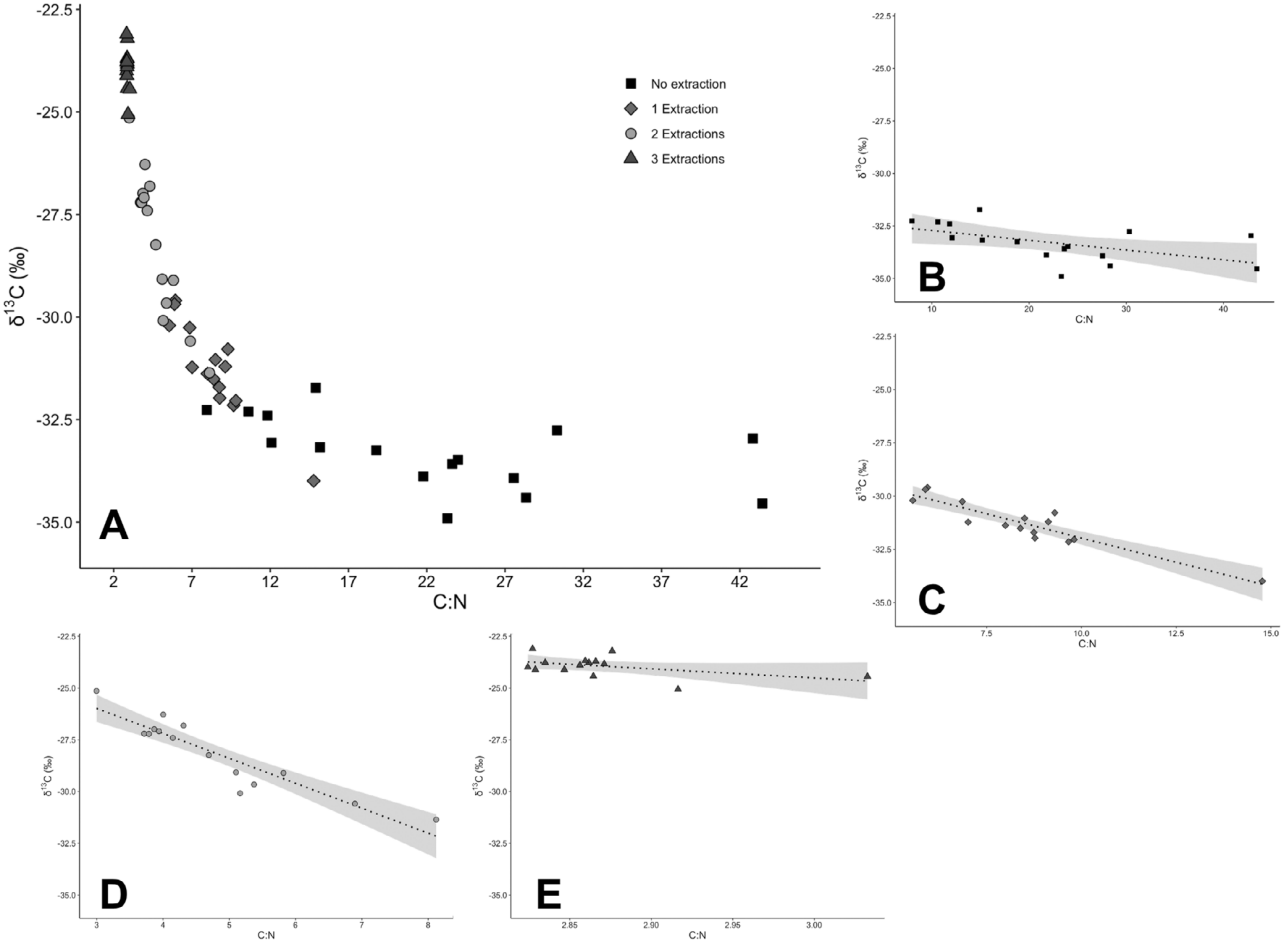
Scatterplots showing the relationship between humpback whale blubber C:N and δ^13^C (*n* = 15) for the control (no extraction) and three treatments (lipid extraction 1, 2, 3) in one plot (A), and in separate plots with the same y‐axis scale for unextracted blubber (B) and blubber after one (C), two (D) and three lipid extractions (E). The dotted line shows the linear regression with confidence intervals.

The δ^13^C of E1 humpback whale blubber varied significantly between the control and all three lipid extraction treatments (pseudo‐*F*_10,37_ = 11.014, *p* = 0.0001). The δ^13^C of the bulk blubber tissue, the control, varied significantly from the δ^13^C of all three treatments, and the δ^13^C of the three treatments also varied significantly from each other, as evidenced by a post hoc pairwise comparison (Table 1). The control had significantly lower δ^13^C than the three lipid extraction treatments. The δ^13^C increased after the first, second, and third lipid extraction (Figure 1). There was a strong, negative, nonlinear association between blubber C:N ratios and blubber δ^13^C (Figure 4). Blubber C:N ratios got progressively smaller with each extraction (Figure 3), and average values were within the range of pure tissues (2.8–3.0) after three lipid extractions (Figure 2, Table 1). The average C:N ratios of E1 humpback whale blubber varied significantly between the control and the three lipid extraction treatments (pseudo‐*F*_10,37_ = 7.7807, *p* = 0.0001). The average blubber C:N ratios of all three lipid extraction treatments were significantly lower in a post hoc pairwise comparison than bulk blubber C:N ratios (Table 1). The average δ^15^N of E1 humpback whale blubber did not vary significantly between bulk tissue, the control, and the three lipid extraction treatments (pseudo‐*F*_10,37_ = 1.0629, *p* = 0.3347) in a PERMANOVA. The average blubber δ^15^N stayed within the range of the expected analytical instrument error of 0.5‰ after the three lipid extraction treatments (Figure 3).

## 4 Discussion

Accounting for how solvents impact δ^13^C and δ^15^N of high lipid content tissues after solvent lipid extraction is becoming increasingly important in cetacean research because of the widespread use of stable isotope analyses in forensic ecology, and rapidly advancing model applications derived from such empirical data. To address this question, we applied a commonly used lipid extraction method to E1 humpback whale blubber and four different, pure protein samples. Results show that the solvents used in the modified Bligh and Dyer (1959) method, methanol and dichloromethane, did not significantly alter the δ^13^C and δ^15^N of the four protein samples after lipid extraction. Each extraction of blubber tissue led to higher δ^13^C, indicating a gradual removal of lipids from the tissue but no significant change in blubber δ^15^N. Blubber C:N ratios were only in the range of pure protein, hence lipid‐free, after three extractions using the modified Bligh and Dyer (1959) method. Our results clearly highlight that methanol and dichloromethane do not inherently alter the δ^13^C of tissues beyond the desired effect of lipid removal; however, they also revealed that lipids are only fully removed from high lipid‐content tissues after multiple extractions using a gentle extraction method. A goal of ‘uniformly low’ lipid concentrations is therefore subject to high uncertainty. Therefore, we advocate for multiple gentle solvent extractions, or alternatively, a stronger solvent extraction technique after validation that the used solvents do not alter δ^13^C beyond the desired effect.

### 4.1 Changes in Pure Protein

To the best of the authors' knowledge, the effects of solvent extraction on tissue δ^13^C have not yet been studied, but knowledge about the effects is critical for stable isotope research. Prior work by Groß et al. (2021) has shown a large disparity between empirical and mathematically calculated tissue lipid content using C:N ratios. The mentioned disparity likely arose from changes in δ^13^C after lipid extraction and not due to varying tissue glycogen content, as δ^13^C decreased with increasing C:N ratios (Groß et al. 2021). Here, we show that the solvents used in the modified Bligh and Dyer (1959) method, dichloromethane and methanol, have no significant inherent effect on δ^13^C after lipid extraction. The majority of δ^13^C of the pure protein tissues stayed within 0.5‰ of the bulk δ^13^C after each lipid extraction, which is the range of the expected instrument error. This means that δ^13^C derived from tissue that has undergone lipid extraction using dichloromethane and methanol can be used for data interpretation without hesitation because δ^13^C do not show more variability than what is expected due to instrument error.

The δ^15^N of all pure protein tissues also remained within the expected instrument error range after each extraction, which is interesting considering that several studies have found a significant increase or decrease in δ^15^N of other tissues following lipid extraction (Clark et al. 2019; Groß et al. 2021; Kiljunen et al. 2006; Kurle and Worthy 2002; Lesage et al. 2010; Post et al. 2007; Ryan et al. 2012; Sotiropoulos et al. 2004). No effect of dichloromethane and methanol on pure protein tissues is indicative of no removal of isotopically light, nitrogenous components such as amino acids. The removal of amino acids is the hypothesised reason for a change in δ^15^N after lipid extraction (Bearhop et al. 2000). However, the extraction method used here has been shown to have little influence on δ^15^N (Logan et al. 2008), which is corroborated by our findings. Potentially, nitrogenous components in the form of amino acids are only removed from tissue alongside lipids but not when no lipids are present in the tissue, and hence removed.

### 4.2 Changes in Humpback Whale Blubber

Solvent lipid extraction methods are commonly used in stable isotope analyses to normalise δ^13^C of high lipid content tissues such as baleen whale blubber because lipids are depleted in ^13^C relative to proteins or carbohydrates, which can lead to misinterpretation of results. Here, we used a gentle lipid extraction technique, the modified Bligh and Dyer (1959) method, because the extraction of lipids for fatty acid analysis was performed on the same samples. In this study, E1 humpback whale blubber δ^13^C increased on average by 10‰ between bulk tissue and the third lipid extraction, while the δ^15^N did not change significantly after each lipid extraction (Table 1). The δ^15^N results are contrary to those found by Groß et al. (2021) for E1 humpback whale skin, which showed a significant increase of δ^15^N after lipid extraction. However, our δ^15^N results align with those found by Ryan et al. (2012) who also did not see a significant change in northern hemisphere humpback whale blubber and skin δ^15^N following lipid extraction. Similar to our δ^13^C results, Ryan et al. (2012) also found an increase in northern hemisphere humpback whale blubber δ^13^C following lipid extraction. The δ^13^C increase they observed averaged 6‰ (Ryan et al. 2012), which is comparable to the 5‰–7‰ increase found in northern fur seal ( *Callorhinus ursinus* ) blubber following lipid extraction (Kurle and Worthy 2002). The 5‰–7‰ increase in blubber δ^13^C in both studies after one lipid extraction is similar to the increase we observed after two lipid extractions.

The different observations regarding δ^13^C and δ^15^N between our study and others may have been caused by tissue composition, the use of different lipid extraction techniques, and solvents used during the extraction. The contrasting δ^15^N trends between E1 humpback whale blubber and skin tissue likely arise due to differences in tissue lipid content because the same extraction method and solvents were used for each tissue (Groß et al. 2021). The higher increase in δ^13^C following lipid extraction observed in our study compared to that observed in Ryan et al.'s (2012) and Kurle and Worthy's (2002) study was likely caused by the use of different lipid extraction techniques. Ryan et al. (2012) used a Soxhlet reflux method with hot *n*‐hexane and acetone for northern hemisphere humpback whales, while Kurle and Worthy (2002) used a Soxhlet extractor with petroleum ether for northern fur seals. Both methods are stronger and more rapid lipid extraction techniques, which is indicated by the average blubber C:N of northern hemisphere humpback whales (2.87) being within the range of pure proteins (2.8–3.0) after one extraction (Ryan et al. 2012). The blubber C:N of northern fur seals were not reported by Kurle and Worthy (2002), which makes it impossible to know whether their lipid extraction method removed all lipids from blubber after one extraction. Our results show that blubber tissue is lipid‐free after three lipid extractions because the average C:N were in the range of pure protein and δ^13^C did not decrease with increasing C:N (Figure 4E).

The increase in blubber δ^13^C in our study following lipid extraction is also substantially higher than the δ^13^C increases observed for lower lipid content tissues such as cetacean skin or muscle, which averaged between 1‰–2‰ (Groß et al. 2021; Lesage et al. 2010; Ryan et al. 2012; Todd et al. 1997). However, reported C:N in the mentioned studies were slightly higher than those of pure protein, ranging from 3.15 to 3.3 (Groß et al. 2021; Lesage et al. 2010; Ryan et al. 2012). This shows that one lipid extraction does not necessarily remove all lipids, even from low lipid‐content tissues. The same was found for other marine mammals. For example, Clark et al. (2019) used a modified Bligh and Dyer (1959) and Folch et al. (1957) method with chloroform and methanol to extract lipids from Pacific walrus ( *Odobenus rosmarus divergens* ) liver, muscle and skin. They reported average skin, liver, and muscle C:N ratios (3.1, 4.0, and 3.3, respectively) above the range of pure proteins after one extraction (Clark et al. 2019). This means that at least one additional lipid extraction would be required to remove all lipids from these liver and muscle tissue samples, despite both having lower lipid content than blubber (Dannenberger et al. 2020). These comparisons to other cetacean and marine mammal species, as well as other tissues, highlight that gentle lipid extraction methods, regardless of the solvent combination used, do not fully extract lipids from the tissue after one extraction, whereas stronger and more rapid lipid extraction approaches, which are being used in many other studies, can achieve that.

We conducted a non‐comprehensive literature review of 40 manuscripts (Table S1) in which the authors used a solvent lipid extraction method on marine mammal tissue prior to δ^13^C and δ^15^N stable isotope analyses. On average, studies included in the review used δ^13^C and δ^15^N stable isotope analyses of one to two tissue types, mainly skin (67.5% of studies), to answer ecological questions about marine mammal species, mainly cetaceans (67.5% of studies; Table S2). Half of the reviewed studies (50%) used the original or a modified Bligh and Dyer (1959) method with Chloroform and Methanol in a 2:1 ratio (80% of studies) to extract lipids from tissues (Tables S1 and S2). The review showed that the majority of studies do not report enough detail to replicate the lipid extraction method or assess its success (Table S1). Some studies did not report which lipid extraction method was used (20% of studies), while others did not report the extraction time (45% of studies), and the majority of studies did not report the amount of solvents used for extraction (75%; Tables S1 and S2). Less than 30% of studies tested the complete removal of lipids from marine mammal tissue prior to conducting stable isotope analyses by either extracting lipids until the supernatant remained clear (12.5% of studies), reporting the C:N ratios before and after the extraction (30% of studies), by reporting the lipid % of the tissues (12.5%), or by at least reporting the C:N ratio after the extraction (57.5% of studies, Table S2). In those studies that reported the C:N ratios after lipid extraction, some C:N ratios were above those of pure proteins (2.8–3.0), which shows that lipids were not completely removed from the tissue, meaning that additional lipid extractions would have been necessary (Table S1). The results from this non‐comprehensive literature review and our study show that we need to consider and account for the effects of solvent lipid extraction on both δ^13^C and δ^15^N when analysing and interpreting results.

## 5 Conclusions

Based on our results, we can advise that the use of the modified Bligh and Dyer (1959) lipid extraction method with dichloromethane and methanol does not alter δ^13^C beyond the desired effect of lipid removal. However, as E1 humpback whale blubber C:N ratios were only in the range of pure proteins after three lipid extractions, we advise that C:N ratios of high lipid content tissues are evaluated after the first lipid extraction and prior to data analysis. If C:N ratios are higher than 3.0, we suggest a second or even third lipid extraction to avoid erroneous interpretations based on depleted ^13^C results, or a stronger solvent extraction technique if the lipid fraction is not of interest, for example, fatty acid analyses. In case δ^13^C and δ^15^N are analysed from the same sample due to cost restraints, we highlight that multiple lipid extractions of one sample may be more costly than the separate analysis of δ^13^C and δ^15^N. As our study only tested the effect of the solvents, dichloromethane and methanol on δ^13^C of pure proteins, using a gentle extraction method, we recommend future studies to conduct similar experiments with other solvents and extraction methods used for lipid normalisation.

## Author Contributions

**J. Groß:** conceptualization (equal), data curation (lead), formal analysis (lead), funding acquisition (supporting), investigation (equal), methodology (equal), project administration (lead), resources (supporting), supervision (supporting), visualization (lead), writing – original draft (lead), writing – review and editing (equal). **B. Fry:** conceptualization (equal), data curation (equal), formal analysis (equal), methodology (equal), supervision (supporting), writing – review and editing (supporting). **J. Eggebo:** data curation (supporting), writing – review and editing (equal). **S. Bengtson Nash:** conceptualization (equal), data curation (equal), funding acquisition (lead), project administration (equal), resources (lead), supervision (lead), writing – original draft (equal), writing – review and editing (equal).

## Funding

The research presented in this manuscript was funded by a Pacific Life Ocean Foundation grant and by the Winifred Violet Scott Trust.

## Conflicts of Interest

The authors declare no conflicts of interest.

## Supporting information


**Table S1:** Table showing the results of the non‐comprehensive literature review. The review included 40 research articles and 24 different categories plus a name and note section. Publications are listed by author name in alphabetical order.
**Table S2:** Table summarising the non‐comprehensive literature review results in percent per category. The literature review included 40 research articles from 1997 to 2023.

## Data Availability

Data used in this manuscript are deposited in the Scientific Committee on Antarctic Research (SCAR) Southern Ocean diet and energetics database (SO‐DIET). The data can be accessed using the following link: https://doi.org/10.5281/zenodo.20047874. It is stored under source publication 429.
