# Supplementary material for: Influence of Solvent Lipid Extraction on Tissue‐Specific Carbon Isotopes
Source: Ecol Evol. 2026 Jun 12;16(6):e73829. doi: 10.1002/ece3.73829 (PMC13261370; doi:10.1002/ece3.73829)
Supplement: Supplementary file 1 — Table S1: Table showing the results of the non‐comprehensive literature review. The review included 40 research articles and 24 different categories plus a name and note section. Publications are listed by author name in alphabetical order. Table S2: Table summarising the non‐comprehensive literature review results in percent per category. The literature review included 40 research articles from 1997 to 2023. [file ECE3-16-e73829-s001.pdf]

Supplementary Materials for

# Influence of Solvent Lipid Extraction on Tissue-Specific Carbon Isotopes

Jasmin Groß *et al*.

*Corresponding author. Email: jasmin.gross@fulbrightmail.org

**This PDF file includes:**

Tables S1 to S2

**Table S1.**

Table showing the results of the non-comprehensive literature review. The review included 40 research articles and 24 different categories plus a name and note section. Publications are listed by author name in alphabetical order.

| | Subject | | | | | Method | | | | | | | |
|---|---|---|---|---|---|---|---|---|---|---|---|---|---|
| Publication | Species | Tissue | N | Cetacean | Mysticete | Method | Time | Amount | Solvent 1 | Solvent 2 | Solvent 3 | Ratio | Number of extractions |
| Arregui et al. 2017 | Common Dolphin | Skin | 10 | Yes | No | / | / | / | Chloroform | Methanol | / | 2:1 | Several rinses |
| | Common Dolphin | Liver | 10 | Yes | No | / | / | / | Chloroform | Methanol | / | 2:1 | Several rinses |
| | Common Dolphin | Kidney | 10 | Yes | No | / | / | / | Chloroform | Methanol | / | 2:1 | Several rinses |
| | Common Dolphin | Muscle | 10 | Yes | No | / | / | / | Chloroform | Methanol | / | 2:1 | Several rinses |
| | Striped Dolphin | Skin | 9 | Yes | No | / | / | / | Chloroform | Methanol | / | 2:1 | Several rinses |
| | Striped Dolphin | Liver | 9 | Yes | No | / | / | / | Chloroform | Methanol | / | 2:1 | Several rinses |
| | Striped Dolphin | Kidney | 9 | Yes | No | / | / | / | Chloroform | Methanol | / | 2:1 | Several rinses |
| | Striped Dolphin | Muscle | 9 | Yes | No | / | / | / | Chloroform | Methanol | / | 2:1 | Several rinses |
| Bas et al. 2019 | South American Sea Lion | Atlas vertebra | 14 | No | No | Folch et al. 1957 | >24h | / | Chloroform | Methanol | Hydrochloric acid | 2:1 | Until supernatant remained clear |
| | South American Sea Lion | Humerus | 14 | No | No | Folch et al. 1957 | >24h | / | Chloroform | Methanol | Hydrochloric acid | 2:1 | Until supernatant remained clear |
| | South American Sea Lion | Basioccipital | 14 | No | No | Folch et al. 1957 | >24h | / | Chloroform | Methanol | Hydrochloric acid | 2:1 | Until supernatant remained clear |
| | Dusky Dolphin | Atlas vertebra | 15 | Yes | No | Folch et al. 1957 | >24h | / | Chloroform | Methanol | Hydrochloric acid | 2:1 | Until supernatant remained clear |
| | Dusky Dolphin | Humerus | 15 | Yes | No | Folch et al. 1957 | >24h | / | Chloroform | Methanol | Hydrochloric acid | 2:1 | Until supernatant remained clear |
| | Dusky Dolphin | Basioccipital | 15 | Yes | No | Folch et al. 1957 | >24h | / | Chloroform | Methanol | Hydrochloric acid | 2:1 | Until supernatant remained clear |
| Borrell et al. 2018 | Fin Whale | Skin | 28 | Yes | Yes | / | / | / | Chloroform | Methanol | / | 2:1 | / |
| Borrell et al. 2021 | Common Dolphin | Skin | 8 | Yes | No | / | / | / | Chloroform | Methanol | / | 2:1 | Several rinses |
| | Striped Dolphin | Skin | 20 | Yes | No | / | / | / | Chloroform | Methanol | / | 2:1 | Several rinses |
| | Bottlenose Dolphin | Skin | 18 | Yes | No | / | / | / | Chloroform | Methanol | / | 2:1 | Several rinses |
| | Risso's Dolphin | Skin | 15 | Yes | No | / | / | / | Chloroform | Methanol | / | 2:1 | Several rinses |
| | Long-finned Pilot Whale | Skin | 3 | Yes | No | / | / | / | Chloroform | Methanol | / | 2:1 | Several rinses |
| | Cuvier's Beaked whale | Skin | 2 | Yes | No | / | / | / | Chloroform | Methanol | / | 2:1 | Several rinses |
| | Fin Whale | Skin | 9 | Yes | Yes | / | / | / | Chloroform | Methanol | / | 2:1 | Several rinses |
| | Bottlenose Dolphin | Skin | 16 | Yes | No | / | / | / | Chloroform | Methanol | / | 2:1 | / |
| Choy et al. 2016 | Beluga | Muscle | 69 | Yes | No | Folch et al. 1957/Bligh and Dyer 1959 | 35min | 0.1-0.2g | Chloroform | Methanol | / | 2:1 | 3 |
| | Beluga | Liver | 68 | Yes | No | Folch et al. 1957/Bligh and Dyer 1959 | 35min | 0.1-0.2g | Chloroform | Methanol | / | 2:1 | 3 |
| Clark et al. 2019 | Walrus | Skin | 35 | No | No | Folch et al. 1957/Bligh and Dyer 1959 | 15min | 0.2 - 0.4g | Chloroform | Methanol | / | 2:1 | Until supernatant remained clear |
| | Walrus | Muscle | 125 | No | No | Folch et al. 1957/Bligh and Dyer 1959 | 15min | 0.2 - 0.4g | Chloroform | Methanol | / | 2:1 | Until supernatant remained clear |
| | Walrus | Bone Collagen | 30 | No | No | Folch et al. 1957/Bligh and Dyer 1959 | 15min | 0.2 - 0.4g | Chloroform | Methanol | / | 2:1 | Until supernatant remained clear |
| | Walrus | Liver | 35 | No | No | Folch et al. 1957/Bligh and Dyer 1959 | 15min | 0.2 - 0.4g | Chloroform | Methanol | / | 2:1 | Until supernatant remained clear |
| Cloyed et al. 2020 | Bottlenose Dolphin | Liver | 9 | Yes | No | Modified Folch et al. 1957 | 15min | / | Chloroform | Methanol | / | 2:1 | Until supernatant remained clear |
| | Bottlenose Dolphin | Muscle | 10 | Yes | No | Modified Folch et al. 1957 | 15min | / | Chloroform | Methanol | / | 2:1 | Until supernatant remained clear |
| | Bottlenose Dolphin | Skin | 10 | Yes | No | Modified Folch et al. 1957 | 15min | / | Chloroform | Methanol | / | 2:1 | Until supernatant remained clear |
| | Manatee | Liver | 7 | No | No | Modified Folch et al. 1957 | 15min | / | Chloroform | Methanol | / | 2:1 | Until supernatant remained clear |
| | Manatee | Muscle | 10 | No | No | Modified Folch et al. 1957 | 15min | / | Chloroform | Methanol | / | 2:1 | Until supernatant remained clear |
| | Manatee | Skin | 9 | No | No | Modified Folch et al. 1957 | 15min | / | Chloroform | Methanol | / | 2:1 | Until supernatant remained clear |
| Cloyed et al. 2021 | Bottlenose Dolphin | Skin | 30 | Yes | No | Modified Folch et al. 1957 | 15 min | 6 mL | Chloroform | Methanol | / | 2:1 | 2-4 times until supernatant remained clear |
| | Bottlenose Dolphin | Muscle | 30 | Yes | No | Modified Folch et al. 1957 | 16 min | 7 mL | Chloroform | Methanol | / | 2:1 | 2-4 times until supernatant remained clear |
| | Bottlenose Dolphin | Liver | 30 | Yes | No | Modified Folch et al. 1957 | 17 min | 8 mL | Chloroform | Methanol | / | 2:1 | 2-4 times until supernatant remained clear |
| Desforges et al. 2022 | Ringed Seal | Muscle | 39 | No | No | Modified Bligh and Dyer 1959 | / | / | Chloroform | Methanol | / | 2:1 | / |
| | Ringed Seal | Liver | 43 | No | No | Modified Bligh and Dyer 1959 | / | / | Chloroform | Methanol | / | 2:1 | / |
| | Harp Seal | Muscle | 8 | No | No | Modified Bligh and Dyer 1959 | / | / | Chloroform | Methanol | / | 2:1 | / |
| | Harp Seal | Liver | 8 | No | No | Modified Bligh and Dyer 1959 | / | / | Chloroform | Methanol | / | 2:1 | / |
| Drago et al. 2015 | South American Sea Lion | Bone | 30 | No | No | Bligh and Dyer 1959 | / | / | Chloroform | Methanol | 0.5 N HCl | 2:1 | / |
| | South American Sea Lion | Skin | 22 | No | No | Bligh and Dyer 1959 | / | / | Chloroform | Methanol | / | 2:1 | / |
| Franco-Trecu et al. 2015 | South American Sea Lion | Blood cell | | No | No | Bligh and Dyer 1959 | / | / | Chloroform | Methanol | / | 2:1 | / |
| | South American Sea Lion | Blood serum | | No | No | Bligh and Dyer 1959 | / | / | Chloroform | Methanol | / | 2:1 | / |
| Fuirst et al. | Atlantic Walrus | Liver | 4 | No | No | Bligh and Dyer 1959 | / | / | Chloroform | Methanol | / | 2:1 | / |
| | Atlantic Walrus | Muscle | 4 | No | No | Bligh and Dyer 1959 | / | / | Chloroform | Methanol | / | 2:1 | / |
| | Narwhal | Liver | 12 | Yes | No | Bligh and Dyer 1959 | / | / | Chloroform | Methanol | / | 2:1 | / |
| | Narwhal | Muscle | 15 | Yes | No | Bligh and Dyer 1959 | / | / | Chloroform | Methanol | / | 2:1 | / |
| | Beluga | Liver | 8 | Yes | No | Bligh and Dyer 1959 | / | / | Chloroform | Methanol | / | 2:1 | / |
| | Beluga | Muscle | 12 | Yes | No | Bligh and Dyer 1959 | / | / | Chloroform | Methanol | / | 2:1 | / |
| Groß et al. 2021 | Humpback Whale | Blubber | 30 | Yes | Yes | Modified Bligh and Dyer 1959 | >12 h | / | Methanol | Dichloromethane | Water | 2:1:0.8 | 1 |
| | Humpback Whale | Skin | 30 | Yes | Yes | Modified Bligh and Dyer 1960 | >12 h | / | Methanol | Dichloromethane | Water | 2:1:0.8 | 1 |
| Horstmann-Dehn et al. 2012 | Bowhead Whale | Muscle | | Yes | Yes | Pinnegar and Polunin 1999 | | 5g | Methanol | Chloroform | Water | 10:5:4 | 1 |
| | Bowhead Whale | Skin | | Yes | Yes | Pinnegar and Polunin 1999 | | 5g | Methanol | Chloroform | Water | 10:5:4 | 1 |
| | Gray Whale | Muscle | | Yes | Yes | Pinnegar and Polunin 1999 | | 5g | Methanol | Chloroform | Water | 10:5:4 | 1 |
| | Gray Whale | Skin | | Yes | Yes | Pinnegar and Polunin 1999 | | 5g | Methanol | Chloroform | Water | 10:5:4 | 1 |
| | Beluga | Muscle | | Yes | No | Pinnegar and Polunin 1999 | | 5g | Methanol | Chloroform | Water | 10:5:4 | 1 |
| | Beluga | Skin | | Yes | No | Pinnegar and Polunin 1999 | | 5g | Methanol | Chloroform | Water | 10:5:4 | 1 |
| Kurle & Worthy 2002 | Fur Seals | Fur | 67 | No | No | Dobush et al. 1985 Soxhlet extractor | 24h | / | Petroleum ether | / | / | / | 1 |
| | Fur Seals | Muscle | 68 | No | No | Dobush et al. 1985 Soxhlet extractor | 24h | / | Petroleum ether | / | / | / | 1 |
| | Fur Seals | Brain | 68 | No | No | Dobush et al. 1985 Soxhlet extractor | 24h | / | Petroleum ether | / | / | / | 1 |
| | Fur Seals | Blubber | 66 | No | No | Dobush et al. 1985 Soxhlet extractor | 24h | / | Petroleum ether | / | / | / | 1 |
| | Fur Seals | Liver | 70 | No | No | Dobush et al. 1985 Soxhlet extractor | 24h | / | Petroleum ether | / | / | / | 1 |
| | Fur Seals | Kidney | 63 | No | No | Dobush et al. 1985 Soxhlet extractor | 24h | / | Petroleum ether | / | / | / | 1 |
| Lesage et al. 2010 | Beluga | Skin | 46 | Yes | No | Folch et al. 1957 | Overnight | 0.2g | Chloroform | Methanol | / | 2:1 | 3 |
| | Harbour Porpoise | Skin | 20 | Yes | No | Folch et al. 1957 | Overnight | 0.2g | Chloroform | Methanol | / | 2:1 | 3 |
| | Minke Whale | Skin | 19 | Yes | Yes | Folch et al. 1957 | Overnight | 0.2g | Chloroform | Methanol | / | 2:1 | 3 |
| | Finback Whale | Skin | 4 | Yes | Yes | Folch et al. 1957 | Overnight | 0.2g | Chloroform | Methanol | / | 2:1 | 3 |
| | Humpback Whale | Skin | 1 | Yes | Yes | Folch et al. 1957 | Overnight | 0.2g | Chloroform | Methanol | / | 2:1 | 3 |
| | Bowhead Whale | Skin | 37 | Yes | Yes | Folch et al. 1957 | Overnight | 0.2g | Chloroform | Methanol | / | 2:1 | 3 |
| Loizaga de Castro et al. 2016 | Dusky Dolphin | Skin | 60 | Yes | No | Bligh and Dyer 1959 | 24h during 5-9 days | / | Chloroform | Methanol | / | 2:1 | Several rinses |
| Loizaga de Castro et al. 2017 | Short-beaked Common Dolphin | Skin | 14 | Yes | No | Bligh and Dyer 1959 | 24h during 5-9 days | / | Chloroform | Methanol | / | 2:1 | Several rinses |
| | Dusky Dolphin | Skin | 13 | Yes | No | Bligh and Dyer 1959 | 24h during 5-9 days | / | Chloroform | Methanol | / | 2:1 | Several rinses |
| Madgett et al. 2019 | Sperm Whale | Blubber | 5 | Yes | No | Modified Bligh and Dyer 1959 according to Hanson and Olley 1963 | / | / | Chloroform | Methanol | Butyl hydroxyl toluene | 2:2 | / |
| | Harbor Seal | Blubber | 10 | No | No | Modified Bligh and Dyer 1959 according to Hanson and Olley 1963 | / | / | Chloroform | Methanol | Butyl hydroxyl toluene | 2:2 | / |
| | Harbour Porpoise | Blubber | 18 | Yes | No | Modified Bligh and Dyer 1959 according to Hanson and Olley 1963 | / | / | Chloroform | Methanol | Butyl hydroxyl toluene | 2:2 | / |
| Mansouri et al. 2019 | Fin Whale | Earplug laminae | 7 | Yes | Yes | Modified Bligh and Dyer 1959 | 40 min | / | Chloroform | Methanol | / | 2:1 | Until supernatant remained clear (3-4 times) |
| | Fin Whale | Earplug laminae | 7 | Yes | Yes | Modified Bligh and Dyer 1959 | 40 min | / | Chloroform | Methanol | / | 2:1 | Until supernatant remained clear (3-4 times) |
| | Blue Whale | Earplug laminae | 7 | Yes | Yes | Modified Bligh and Dyer 1959 | 40 min | / | Chloroform | Methanol | / | 2:1 | Until supernatant remained clear (3-4 times) |
| | Blue Whale | Earplug laminae | 7 | Yes | Yes | Modified Bligh and Dyer 1959 | 40 min | / | Chloroform | Methanol | / | 2:1 | Until supernatant remained clear (3-4 times) |
| | Humpback Whale | Earplug laminae | 7 | Yes | Yes | Modified Bligh and Dyer 1959 | 40 min | / | Chloroform | Methanol | / | 2:1 | Until supernatant remained clear (3-4 times) |
| | Humpback Whale | Earplug laminae | 7 | Yes | Yes | Modified Bligh and Dyer 1959 | 40 min | / | Chloroform | Methanol | / | 2:1 | Until supernatant remained clear (3-4 times) |
| Neves et al. 2023 | Atlantic Spotted Dolphin | Muscle | 8 | Yes | No | / | / | / | Chloroform | Methanol | / | 2:1 | / |
| | Franciscana Dolphin | Muscle | 15 | Yes | No | / | / | / | Chloroform | Methanol | / | 2:1 | / |
| | Guiana Dolphin | Muscle | 48 | Yes | No | / | / | / | Chloroform | Methanol | / | 2:1 | / |
| | Rough-toothed Dolphin | Muscle | 5 | Yes | No | / | / | / | Chloroform | Methanol | / | 2:1 | / |
| Newsome et al. 2018 | Common Dolphin | Skin | 30 | Yes | No | / | 24h | / | Chloroform | Methanol | / | 2:1 | 3 |
| | Common Dolphin | Skin | 51 | Yes | No | / | 24h | / | Chloroform | Methanol | / | 2:1 | 3 |
| | Bottlenose Dolphin | Skin | 4 | Yes | No | / | 24h | / | Chloroform | Methanol | / | 2:1 | 3 |
| | Risso's Dolphin | Skin | 3 | Yes | No | / | 24h | / | Chloroform | Methanol | / | 2:1 | 3 |
| | Pacific White-sided Dolphin | Skin | 1 | Yes | No | / | 24h | / | Chloroform | Methanol | / | 2:1 | 3 |
| | Northern Right-whale Dolphin | Skin | 1 | Yes | No | / | 24h | / | Chloroform | Methanol | / | 2:1 | 3 |
| Nicholson et al. 2021 | Indo-Pacific Bottlenose Dolphin | Blubber or Skin | 96 | Yes | No | / | 24h | / | Chloroform | Methanol | / | 2:1 | 3 |
| Ogilvy et al. 2023 | Hector's Dolphin | Skin | 111 | Yes | No | / | / | / | Chloroform | Methanol | / | 2:1 | / |
| Payo-Payo et al. 2013 | Striped Dolphin | Muscle | 3 | Yes | No | Bligh and Dyer 1959 | / | / | Chloroform | Methanol | / | 2:1 | / |
| | Striped Dolphin | Skin | 3 | Yes | No | Bligh and Dyer 1959 | / | / | Chloroform | Methanol | / | 2:1 | / |
| Peters et al. 2020 | Common Dolphin | Skin | 56 | Yes | No | DIONEX 200 accelerated solvent extraction system | 5 min | / | Dichloromethane | / | / | 2:1 | 3 |
| Pirotta et al. 2020 | Sperm Whale | Skin | 20 | Yes | No | Bligh and Dyer 1959 | / | / | Chloroform | Methanol | / | 2:1 | 1 |
| Polito and Goebel 2010 | Antarctic Fur Seal | Milk | 67 | No | No | Modified Folch et al. 1957 | 15 min & 30 sec | 2.5 ml | Chloroform | Methanol | NaCl Solution | 1:1.5 | 1 |
| Ruiz-Cooley et al. 2004 | Sperm Whale | Skin | 43 | Yes | No | Modified Bligh and Dyer 1959 Sonication | / | / | Chloroform | Methanol | / | 1:1 | 1 |
| Ruiz-Cooley et al. 2012 | Sperm Whale | Skin | 71 | Yes | No | Modified Bligh and Dyer 1959 Sonication | / | / | Chloroform | Methanol | / | 2:1 | 2 |
| Ryan et al. 2012 | Fin Whale | Skin | 22 | Yes | Yes | Soxhlet reflux | 12h | / | *n*-hexane | Acetone | / | 1:1 | 1 |
| | Fin Whale | Blubber | 32 | Yes | Yes | Soxhlet reflux | 12h | / | *n*-hexane | Acetone | / | 1:1 | 1 |
| | Humpback Whale | Skin | 6 | Yes | Yes | Soxhlet reflux | 12h | / | *n*-hexane | Acetone | / | 1:1 | 1 |
| | Humpback Whale | Blubber | 22 | Yes | Yes | Soxhlet reflux | 12h | / | *n*-hexane | Acetone | / | 1:1 | 1 |
| | Minke Whale | Skin | 32 | Yes | Yes | Soxhlet reflux | 12h | / | *n*-hexane | Acetone | / | 1:1 | 1 |
| | Minke Whale | Blubber | 6 | Yes | Yes | Soxhlet reflux | 12h | / | *n*-hexane | Acetone | / | 1:1 | 1 |
| Ryan et al. 2014 | Humpback Whale | Skin | 4 | Yes | Yes | Soxhlet reflux | 24h | 150 ml | n-hexane | Acetone | / | 1:1 | 1 |
| | Fin Whale | Skin | 21 | Yes | Yes | Soxhlet reflux | 24h | 150 ml | n-hexane | Acetone | / | 1:1 | 1 |
| Silva et al. 2019 | Blue Whale | Skin | 17 | Yes | Yes | / | / | / | Chloroform | Methanol | / | 2:1 | 1 |
| | Blue Whale | Faeces | 1 | Yes | Yes | / | / | / | Chloroform | Methanol | / | 2:1 | 1 |
| | Fin Whale | Skin | 42 | Yes | Yes | / | / | / | Chloroform | Methanol | / | 2:1 | 1 |
| | Fin Whale | Faeces | 10 | Yes | Yes | / | / | / | Chloroform | Methanol | / | 2:1 | 1 |
| | Sei Whale | Skin | 36 | Yes | Yes | / | / | / | Chloroform | Methanol | / | 2:1 | 1 |
| | Sei Whale | Faeces | 2 | Yes | Yes | / | / | / | Chloroform | Methanol | / | 2:1 | 1 |
| Smith et al. 2020 | Sowerby's Beaked Whale | Kidney | 18 | Yes | No | / | 30min | / | Chloroform | Methanol | / | 2:1 | 1 |
| | Sowerby's Beaked Whale | Kidney | 18 | Yes | No | / | 30min | / | Cyclohexane | / | / | 2:1 | 1 |
| | Sowerby's Beaked Whale | Liver | 17 | Yes | No | / | 30min | / | Chloroform | Methanol | / | 2:1 | 1 |
| | Sowerby's Beaked Whale | Liver | 17 | Yes | No | / | 30min | / | Cyclohexane | / | / | 2:1 | 1 |
| | Sowerby's Beaked Whale | Muscle | 18 | Yes | No | / | 30min | / | Chloroform | Methanol | / | 2:1 | 1 |
| | Sowerby's Beaked Whale | Muscle | 18 | Yes | No | / | 30min | / | Cyclohexane | / | / | 2:1 | 1 |
| | Sowerby's Beaked Whale | Skin | 24 | Yes | No | / | 30min | / | Chloroform | Methanol | / | 2:1 | 1 |
| | Sowerby's Beaked Whale | Skin | 24 | Yes | No | / | 30min | / | Cyclohexane | / | / | 2:1 | 1 |
| Tatsch et al. 2016 | Cuvier's Beaked Whale | Bone Collagen | 4 | Yes | No | Modified Bligh and Dyer 1959 Soxhlet reflux | 24h | 0.001g | Chloroform | Methanol | / | 2:1 | 1 |
| | Arnouz's Beaked Whale | Bone Collagen | 3 | Yes | No | Modified Bligh and Dyer 1959 Soxhlet reflux | 24h | 0.001g | Chloroform | Methanol | / | 2:1 | 1 |
| | Blainville's Beaked Whale | Bone Collagen | 2 | Yes | No | Modified Bligh and Dyer 1959 Soxhlet reflux | 24h | 0.001g | Chloroform | Methanol | / | 2:1 | 1 |
| | Gray's Beaked Whale | Bone Collagen | 2 | Yes | No | Modified Bligh and Dyer 1959 Soxhlet reflux | 24h | 0.001g | Chloroform | Methanol | / | 2:1 | 1 |
| | Layard's Beaked Whale | Bone Collagen | 1 | Yes | No | Modified Bligh and Dyer 1959 Soxhlet reflux | 24h | 0.001g | Chloroform | Methanol | / | 2:1 | 1 |
| | Hector's Beaked Whale | Bone Collagen | 1 | Yes | No | Modified Bligh and Dyer 1959 Soxhlet reflux | 24h | 0.001g | Chloroform | Methanol | / | 2:1 | 1 |
| Todd et al. 1997 | Humpback Whale | Muscle | 4 | Yes | Yes | Sonication and Soxhlet | 3h | / | Dichloromethane | Methanol | / | 6.7:1 | 1 |
| | Humpback Whale | Skin | 4 | Yes | Yes | Sonication | 3h | / | Dichloromethane | Methanol | / | 6.7:1 | 1 |
| | Humpback Whale | Skin | 4 | Yes | Yes | Soxhlet | 3h | / | Dichloromethane | Methanol | / | 6.7:1 | 1 |
| | Humpback Whale | Blubber | 4 | Yes | Yes | Sonication and Soxhlet | 3h | / | Dichloromethane | Methanol | / | 6.7:1 | 1 |
| Wild et al. 2018 | Sperm Whale | Skin | 28 | Yes | No | / | 20 min | 2 ml | Chloroform | Methanol | / | 2:1 | 3 |
| | Humpback Whale | Skin | 1 | Yes | Yes | / | 20 min | 2 ml | Chloroform | Methanol | / | 2:1 | 3 |
| | Fin Whale | Skin | 1 | Yes | Yes | / | 20 min | 2 ml | Chloroform | Methanol | / | 2:1 | 3 |
| Wilson et al. 2014 | Bottlenose Dolphin | Skin | 10 | Yes | No | Sweeting et al. 2006 (cold) | 1h | / | Chloroform | Methanol | Water | 2:1 | 2 |
| | Bottlenose Dolphin | Skin | 10 | Yes | No | Post et al. 2007 (hot) | / | / | Chloroform | Methanol | NaCl Solution | 2.13:1:1.7 | 1 |
| | Bottlenose Dolphin | Skin | 10 | Yes | No | Modified Sweeting et al. 2006 | / | / | Chloroform | Methanol | / | 2:1 | 1 |
| Young et al. 2014 | Ringed Seal | Muscle | 377 | No | No | Bligh and Dyer 1959 | 24h | 5 ml | Chloroform | Methanol | / | 2:1 | 2 |
| | Ringed Seal | Hair | 377 | No | No | / | / | / | / | / | / | / | / |
| Yurkowski et al. 2015 | Bearded Seal | Liver | 18 | No | No | Bligh and Dyer 1959 | / | / | Chloroform | Methanol | / | 2:1 | 1 |
| | Bearded Seal | Muscle | 22 | No | No | Bligh and Dyer 1959 | / | / | Chloroform | Methanol | / | 2:1 | 1 |
| | Harbor Seal | Liver | 11 | No | No | Bligh and Dyer 1959 | / | / | Chloroform | Methanol | / | 2:1 | 1 |
| | Harbor Seal | Muscle | 11 | No | No | Bligh and Dyer 1959 | / | / | Chloroform | Methanol | / | 2:1 | 1 |
| | Harp Seal | Liver | 7 | No | No | Bligh and Dyer 1959 | / | / | Chloroform | Methanol | / | 2:1 | 1 |
| | Harp Seal | Muscle | 7 | No | No | Bligh and Dyer 1959 | / | / | Chloroform | Methanol | / | 2:1 | 1 |
| | Ringed Seal | Liver | 44 | No | No | Bligh and Dyer 1959 | / | / | Chloroform | Methanol | / | 2:1 | 1 |
| | Ringed Seal | Muscle | 58 | No | No | Bligh and Dyer 1959 | / | / | Chloroform | Methanol | / | 2:1 | 1 |
| | Walrus | Liver | 20 | No | No | Bligh and Dyer 1959 | / | / | Chloroform | Methanol | / | 2:1 | 1 |
| | Walrus | Muscle | 19 | No | No | Bligh and Dyer 1959 | / | / | Chloroform | Methanol | / | 2:1 | 1 |
| | Beluga | Liver | 29 | Yes | No | Bligh and Dyer 1959 | / | / | Chloroform | Methanol | / | 2:1 | 1 |
| | Beluga | Muscle | 31 | Yes | No | Bligh and Dyer 1959 | / | / | Chloroform | Methanol | / | 2:1 | 1 |
| | Bowhead Whale | Muscle | 7 | Yes | Yes | Bligh and Dyer 1959 | / | / | Chloroform | Methanol | / | 2:1 | 1 |
| | Narwhal | Liver | 5 | Yes | No | Bligh and Dyer 1959 | / | / | Chloroform | Methanol | / | 2:1 | 1 |
| | Narwhal | Muscle | 3 | Yes | No | Bligh and Dyer 1959 | / | / | Chloroform | Methanol | / | 2:1 | 1 |
| | Narwhal | Skin | 39 | Yes | No | Bligh and Dyer 1959 | / | / | Chloroform | Methanol | / | 2:1 | 1 |
| Yurkowski et al. 2018 | Beluga | Muscle | 72 | Yes | No | Modified Bligh and Dyer 1967 | / | / | Chloroform | Methanol | / | 2:1 | / |
| | Ringed Seal | Muscle | 228 | No | No | Modified Bligh and Dyer 1968 | / | / | Chloroform | Methanol | / | 2:1 | / |
| Yurkowski et al. 2023 | Atlantic Walrus | Muscle | 4 | No | No | Modified Bligh and Dyer 1959 | / | / | Chloroform | Methanol | / | 2:1 | / |
| | Atlantic Walrus | Liver | 4 | No | No | Modified Bligh and Dyer 1960 | / | / | Chloroform | Methanol | / | 2:1 | / |
| | Narwhal | Muscle | 15 | Yes | No | Modified Bligh and Dyer 1961 | / | / | Chloroform | Methanol | / | 2:1 | / |
| | Narwhal | Liver | 12 | Yes | No | Modified Bligh and Dyer 1962 | / | / | Chloroform | Methanol | / | 2:1 | / |
| | Beluga | Muscle | 20 | Yes | No | Modified Bligh and Dyer 1963 | / | / | Chloroform | Methanol | / | 2:1 | / |
| | Beluga | Liver | 8 | Yes | No | Modified Bligh and Dyer 1964 | / | / | Chloroform | Methanol | / | 2:1 | / |
| | Ringed Seal | Muscle | 41 | No | No | Modified Bligh and Dyer 1965 | / | / | Chloroform | Methanol | / | 2:1 | / |
| | Ringed Seal | Liver | 45 | No | No | Modified Bligh and Dyer 1966 | / | / | Chloroform | Methanol | / | 2:1 | / |

Results

| Test for complete extraction | before Extration | CN after Extration | CN Decrease | $\delta^{13}$C before Extration | $\delta^{13}$C after Extration | Increase | Significant? | before Extration | Extraction | increase | $\delta^{15}$N decrease | Significant? | % Lipid | Notes |
|---|---|---|---|---|---|---|---|---|---|---|---|---|---|---|
| No | / | 3.16 ± 0.61 | / | / | -17.75 ± 0.12 | / | / | / | 12.38 ± 0.07 | / | / | / | / | Powdered tissue was rinsed with the lipid extration solvents several times |
| No | / | 3.16 ± 0.61 | / | / | -17.56 ± 0.28 | / | / | / | 13.4 ± 0.62 | / | / | / | / | Mean of all skin position values is reported heren |
| No | / | 3.16 ± 0.61 | / | / | -17.73 ± 0.54 | / | / | / | 13.30 ± 0.59 | / | / | / | / | |
| No | / | 3.16 ± 0.61 | / | / | -17.71 ± 0.45 | / | / | / | 12.55 ± 0.63 | / | / | / | / | |
| No | / | 3.16 ± 0.61 | / | / | -17.82 ± 0.13 | / | / | / | 10.47 ± 0.29 | / | / | / | / | |
| No | / | 3.16 ± 0.61 | / | / | -17.51 ± 0.45 | / | / | / | 11.0 ± 0.5 | / | / | / | / | |
| No | / | 3.16 ± 0.61 | / | / | -17.54 ± 0.2 | / | / | / | 11.18 ± 0.46 | / | / | / | / | |
| No | / | 3.16 ± 0.61 | / | / | -17.73 ± 0.33 | / | / | / | 10.16 ± 0.38 | / | / | / | / | |
| Yes | 3.7 | 3.3 | Yes | -13.6 | -13.3 | Yes | No | 22.3 | 22.1 | No | Yes | No | 8.9 | For $\delta^{15}$N, significance values were only available for all bones combined |
| Yes | 3.6 | 3.2 | Yes | -12.9 | -12.6 | Yes | No | 22.4 | 21.9 | No | Yes | No | 10.4 | |
| Yes | 3.8 | 3.2 | Yes | -13.2 | -12.5 | Yes | Yes | 21.9 | 23.2 | Yes | No | No | 0.7 | |
| Yes | 3.9 | 3.5 | Yes | -14.4 | -14.1 | Yes | No | 18.6 | 19.0 | Yes | No | No | 7.2 | |
| Yes | 4.6 | 3.5 | Yes | -15.8 | -14.4 | Yes | Yes | 18.8 | 19.2 | Yes | No | No | 11.8 | |
| Yes | 4.1 | 3.4 | Yes | -15.1 | -13.0 | Yes | Yes | 18.5 | 19.3 | Yes | No | No | 1.2 | |
| No | / | / | / | / | -19.41 | / | / | / | 9.4 | / | / | / | / | |
| No | / | / | / | / | -17.8 | / | / | / | 10.7 | / | / | / | / | |
| No | / | / | / | / | -18.2 | / | / | / | 10.6 | / | / | / | / | |
| No | / | / | / | / | -16.8 | / | / | / | 12.9 | / | / | / | / | |
| No | / | / | / | / | -16.8 | / | / | / | 13.1 | / | / | / | / | |
| No | / | / | / | / | -17.1 | / | / | / | 13.1 | / | / | / | / | |
| No | / | / | / | / | -16.3 | / | / | / | 13.8 | / | / | / | / | |
| No | / | / | / | / | -19.4 | / | / | / | 9.1 | / | / | / | / | |
| Yes | / | 3.08 | / | / | -18.9 | / | / | / | 14.0 | / | / | / | / | |
| No | 3.4 | 3.1 | Yes | -19.0 | -18.8 | Yes | No | 17.2 | 17.4 | Yes | No | No | 9.3 | |
| No | 4.5 | 3.4 | Yes | -20.9 | -19.4 | Yes | No | 18.1 | 18.3 | Yes | No | No | 13.9 | |
| No | 4.1 | 3.1 | Yes | -16.7 | -14.8 | Yes | Yes | 14.7 | 14.8 | Yes | No | No | / | |
| No | 3.5 | 3.3 | Yes | -17.0 | -16.8 | Yes | No | 12.6 | 12.6 | No | Yes | Yes | / | |
| No | 2.9 | / | / | -14.8 | / | / | / | 12.2 | / | / | / | / | / | |
| No | 4.7 | 4 | Yes | -18.3 | -17.3 | Yes | Yes | 12.6 | 12.8 | Yes | No | Yes | / | |
| No | 4.48 | 3.26 | Yes | -19.64 | -17.61 | Yes | Yes | 15.94 | 15.63 | No | Yes | No | / | |
| No | 3.36 | 3.17 | Yes | -18.34 | -17.97 | Yes | Yes | 14.3 | 14.58 | Yes | No | No | / | |
| No | 4.4 | 3.11 | Yes | -19.98 | -18.27 | Yes | Yes | 14.66 | 14.79 | Yes | No | No | / | |
| No | 4.24 | 3.42 | Yes | -19.99 | -19.63 | Yes | No | 8.79 | 8.54 | No | Yes | No | / | |
| No | 3.46 | 3.25 | Yes | -19.88 | -19.57 | Yes | No | 7.24 | 7.66 | Yes | No | No | / | |
| No | 3.74 | 3.34 | Yes | -20.7 | -20.47 | Yes | No | 8.51 | 8.59 | Yes | No | No | / | |
| Yes | / | / | / | / | / | / | / | / | / | / | / | / | / | |
| Yes | / | / | / | / | / | / | / | / | / | / | / | / | / | |
| Yes | / | / | / | / | / | / | / | / | / | / | / | / | / | |
| / | / | / | / | / | -19.8 | / | / | / | 16.3 | / | / | / | / | |
| / | / | / | / | / | -19.6 | / | / | / | 16.9 | / | / | / | / | |
| / | / | / | / | / | -19.6 | / | / | / | 15.1 | / | / | / | / | |
| / | / | / | / | / | -19.6 | / | / | / | 16.2 | / | / | / | / | |
| No | / | / | / | / | -12.23 ± 0.21 | / | / | / | 20.75 ± 0.57 | / | / | / | / | 0.5 N HCl for 24h for decarbonisation just for $\delta^{13}$C and lipid extraction only for $\delta^{13}$C |
| No | / | / | / | / | -14.05 ± 0.21 | / | / | / | 20.85 ± 1.49 | / | / | / | / | $\delta^{15}$N values were determined without lipid extraction |
| No | / | / | / | / | 3.9 ± 0.16 | / | / | / | 7.69 ± 0.18 | / | / | / | / | |
| No | / | / | / | / | 0.3 ± 0.01 | / | / | / | 12.1 ± 0.27 | / | / | / | / | |
| No | / | 3.9 | / | / | -18.5 | / | / | / | 9.8 | / | / | / | / | |
| No | / | 3.1 | / | / | -17.2 | / | / | / | 10.4 | / | / | / | / | |
| No | / | 3.3 | / | / | -18.3 | / | / | / | 16.3 | / | / | / | / | |
| No | / | 3.1 | / | / | -18.1 | / | / | / | 15.3 | / | / | / | / | |
| No | / | 3.4 | / | / | -18.3 | / | / | / | 17 | / | / | / | / | |
| No | / | 3.1 | / | / | -18.2 | / | / | / | 16 | / | / | / | / | |
| Yes | / | 3.96 | / | / | -26.36 | / | / | / | 7.19 | / | / | / | / | |
| Yes | 3.69 | 3.19 | Yes | -26.97 | -25.57 | Yes | Yes | 6.15 | 6.83 | Yes | No | Yes | / | |
| No | / | / | / | -20.63 | -20.27 | Yes | Yes | 13.29 | 13.33 | Yes | No | No | / | Skin significantly enriched in d15n compared to muscle prior to extraction |
| No | / | / | / | -20.97 | -20.49 | Yes | Yes | 13.63 | 13.81 | Yes | No | No | / | Skin signifcantly depleted in d13c compared to muscle prior to extraction |
| No | / | / | / | -17.32 | / | / | / | 12.03 | / | / | / | / | / | |
| No | / | / | / | -17.62 | -16.72 | Yes | Yes | 13.21 | 13.34 | Yes | No | No | / | |
| No | / | / | / | -18.41 | -18.26 | Yes | No | 16.74 | 16.7 | No | Yes | No | / | |
| No | / | / | / | -19.3 | -18.67 | Yes | Yes | 16.81 | 16.75 | No | Yes | No | / | |
| No | / | / | / | / | -17.65 ± 0.21 | / | / | / | 14.85 ± 0.07 | / | / | / | / | |
| No | / | / | / | / | -18.05 ± 0.07 | / | / | / | 15.35 ± 0.35 | / | / | / | / | |
| No | / | / | / | / | -18.25 ± 0.21 | / | / | / | 16.95 ± 0.07 | / | / | / | / | |
| No | / | / | / | -24.65 ± 0.07 | -18.9 ± 1.13 | Yes | Yes | 15.9 ± 0.14 | 17.1 ± 0.1 | Yes | No | Yes | / | |
| No | / | / | / | / | -18.3 ± 0.14 | / | / | / | 16.1 ± 0.14 | / | / | / | / | |
| No | / | / | / | / | -18.35 ± 0.21 | / | / | / | 16.35 ± 0.07 | / | / | / | / | |
| Yes | / | / | / | -18.1 | -16.4 | Yes | Yes | 16.3 | 16.5 | Yes | No | Yes | / | |
| Yes | / | / | / | -20.1 | -18.0 | Yes | Yes | 14.4 | 14.7 | Yes | No | Yes | / | |
| Yes | / | / | / | -20.9 | -18.3 | Yes | Yes | 13.2 | 13.1 | No | Yes | Yes | / | |
| Yes | / | / | / | -20.9 | -18.3 | Yes | Yes | 13.2 | 13.1 | No | Yes | Yes | / | |
| Yes | / | / | / | -20.9 | -18.3 | Yes | Yes | 13.2 | 13.1 | No | Yes | Yes | / | |
| Yes | / | / | / | / | -19.5 | / | / | / | 13.2 | / | / | / | / | |
| Yes | / | <4 | / | / | -16.23 ± 0.39 | / | / | / | 18.38 ± 0.44 | / | / | / | / | |
| Yes | / | <4 | / | / | -16.2 | / | / | / | 19.2 | / | / | / | / | |
| Yes | / | <4 | / | / | -16.6 | / | / | / | 18.3 | / | / | / | / | |
| No | / | / | / | / | -14.6 | / | / | / | 13.36 | / | / | / | / | |
| No | / | / | / | / | -16.36 | / | / | / | 16.62 | / | / | / | / | |
| No | / | / | / | / | -16.48 | / | / | / | 17.69 | / | / | / | / | |
| Yes | 13.5 | 4.9 | Yes | -22.4 | -18.3 | Yes | Yes | 9.0 | 8.7 | No | Yes | Yes | / | |
| Yes | 9.0 | 5.3 | Yes | -27.5 | -25.7 | Yes | Yes | 6.2 | 5.9 | No | Yes | Yes | / | |
| Yes | 7.6 | 4.7 | Yes | -28.1 | -25.9 | Yes | Yes | 6.1 | 6.0 | No | Yes | Yes | / | |
| Yes | 8.9 | 4.6 | Yes | -26.9 | -23.8 | Yes | Yes | 6.9 | 6.3 | No | Yes | Yes | / | |
| Yes | 7.5 | 4.7 | Yes | -19.8 | -18.6 | Yes | Yes | 13.4 | 13.2 | No | Yes | Yes | / | |
| Yes | 13.6 | 5.2 | Yes | -20.5 | -18.3 | Yes | Yes | 12.6 | 12.2 | No | Yes | Yes | / | |
| Yes | / | / | / | / | -17.2 | / | / | / | 13.7 | / | / | / | / | Mathematical lipid correction applied for samples with C:N >3.5 after extraction |
| Yes | / | / | / | / | -17.3 | / | / | / | 14.4 | / | / | / | / | |
| Yes | / | / | / | / | -16.9 | / | / | / | 14.2 | / | / | / | / | |
| Yes | / | / | / | / | -16.3 | / | / | / | 15.1 | / | / | / | / | |
| Yes | / | / | / | / | / | / | / | / | / | / | / | / | / | After lipid extraction rinsed 5x with deionized water and lyophilized |
| Yes | / | / | / | / | / | / | / | / | / | / | / | / | / | |
| Yes | / | / | / | / | / | / | / | / | / | / | / | / | / | |
| Yes | / | / | / | / | / | / | / | / | / | / | / | / | / | |
| Yes | / | / | / | / | / | / | / | / | / | / | / | / | / | |
| Yes | / | / | / | / | / | / | / | / | / | / | / | / | / | |
| No | / | / | / | / | -29.07 | / | / | 11.13 | / | / | / | / | / | Rinsed with deionised water |
| Yes | / | 3-4 | / | / | -17.1 | / | / | / | 15.1 | / | / | / | / | Lipid extraction was deemed successful if the C:N ratio was between 3.0 and 4.0 |
| No | / | / | / | / | -17.53 ± 0.08 | / | / | / | 9.74 ± 0.008 | / | / | / | / | |
| No | / | / | / | / | -17.02 ± 0.19 | / | / | / | 11.02 ± 0.23 | / | / | / | / | |
| No | >3.5 | <3.5 | / | / | -16.53 ± 0.05 | / | / | 14.85 ± 0.05 | / | / | / | / | / | 11 samples were lipid-extracted and the others mathematically corrected using the lipid correction factor from the extracted samples |
| / | / | 3.1 - 3.6 | / | / | -18.2 ± 0.28 | / | / | / | 11.8 ± 0.85 | / | / | / | / | |
| / | / | 3.5 ± 0.11 | / | / | -24.27 ± 1.1 | / | / | / | 10.18 ± 0.92 | / | / | / | 27.48 ± 3.87 | |
| No | / | 3.3 ± 0.26 | / | / | -13.83 ± 0.06 | / | / | / | 19.63 ± 0.29 | / | / | / | / | |
| No | / | / | / | / | -16.55 ± 0.13 | / | / | / | 12.3 ± 0.2 | / | / | / | / | Some samples were extratec with a 1:1 solvent ration and some with a 2:1 ratio. Both methods were compared. |
| No | 5.57 | 3.67 | Yes | -20.2 | -18.2 | Yes | Yes | 12.0 | 11.9 | No | Yes | No | / | |
| No | 18.61 | 3.15 | Yes | -22.1 | -15.4 | Yes | Yes | 11.6 | 12.8 | Yes | No | Yes | / | |
| No | 4.49 | 3.3 | Yes | -21.3 | -19.6 | Yes | Yes | 12.8 | 12.9 | Yes | No | No | / | |
| No | 10.98 | 2.87 | Yes | -23.2 | -17.3 | Yes | Yes | 13.5 | 13.7 | Yes | No | No | / | |
| No | 7.35 | 3.24 | Yes | -21.8 | -17.9 | Yes | Yes | 13.8 | 13.8 | No | Yes | Yes | / | |
| No | 22.22 | 3.06 | Yes | -25.0 | -16.9 | Yes | / | 12.7 | 14.9 | Yes | No | / | / | |
| No | / | 3.49 | / | / | -17.8 | / | / | / | 12.9 | / | / | / | / | |
| No | / | 3.68 | / | / | -18.2 | / | / | / | 12.1 | / | / | / | / | |
| No | / | / | / | / | -18.68 | / | / | / | 9.1 | / | / | / | / | |
| No | / | / | / | / | -19.78 | / | / | / | 5.3 | / | / | / | / | |
| No | / | / | / | / | -19.44 | / | / | / | 9.54 | / | / | / | / | |
| No | / | / | / | / | -20.61 | / | / | / | 6.16 | / | / | / | / | |
| No | / | / | / | / | -17.5 | / | / | / | 9.01 | / | / | / | / | |
| No | / | / | / | / | -20.68 | / | / | / | 4.78 | / | / | / | / | |
| No | 3.3 | 3.2 | Yes | -18.0 | -17.7 | Yes | No | 13.1 | 13.3 | Yes | No | No | / | Significant differences in d13c values between two extration methods |
| No | 3.3 | 3.3 | No | -18.0 | -18.0 | No | No | 13.1 | 13.1 | No | Yes | No | / | Significant difference in d15n values between two methods only in kidney |
| No | 3.4 | 3.2 | Yes | -18.1 | -17.8 | Yes | Yes | 13.3 | 13.2 | No | Yes | No | / | Samples all had been preserved in ethanol prior to analysis |
| No | 3.4 | 3.4 | No | -18.1 | -18.2 | No | No | 13.3 | 13.2 | No | Yes | No | / | |
| No | 3.7 | 3.2 | Yes | -18.9 | -18.1 | Yes | Yes | 12.4 | 12.6 | Yes | No | No | / | |
| No | 3.7 | 3.3 | No | -18.9 | -18.3 | Yes | No | 12.4 | 12.7 | Yes | No | No | / | |
| No | 6.4 | 3.8 | Yes | -21.1 | -19.1 | Yes | Yes | 12.2 | 12.7 | Yes | No | No | / | |
| No | 6.4 | 4.1 | Yes | -21.1 | -19.5 | Yes | Yes | 12.2 | 12.6 | Yes | No | No | / | |
| No | 4.61 | 3.84 | Yes | -18.0 | -16.8 | Yes | Yes | 13.6 | 13.8 | Yes | No | / | / | |
| No | 4.61 | 3.84 | Yes | -18.0 | -16.8 | Yes | Yes | 13.6 | 13.8 | Yes | No | / | / | |
| No | 4.61 | 3.84 | Yes | -18.0 | -16.8 | Yes | Yes | 13.6 | 13.8 | Yes | No | / | / | |
| No | 4.61 | 3.84 | Yes | -18.0 | -16.8 | Yes | Yes | 13.6 | 13.8 | Yes | No | / | / | |
| No | 4.61 | 3.84 | Yes | -18.0 | -16.8 | Yes | Yes | 13.6 | 13.8 | Yes | No | / | / | |
| No | 4.61 | 3.84 | Yes | -18.0 | -16.8 | Yes | Yes | 13.6 | 13.8 | Yes | No | / | / | |
| No | / | / | / | -19.1 | / | / | / | / | / | / | / | / | / | No clear table with results, only figures and in-text mention of some values |
| No | / | / | / | -19.8 | -18.4 | Yes | / | / | / | / | / | / | / | |
| No | / | / | / | -19.8 | -18.6 | Yes | / | / | / | / | / | / | / | |
| No | / | / | / | -23.7 | / | / | / | / | / | / | / | / | / | |
| No | 3.3-4.7 | 3.4 | Yes | / | -16.8 | / | Yes | / | 17.2 | / | / | No | / | |
| No | / | 3.3 | / | / | 17.5 | / | / | / | 12.4 | / | / | / | / | |
| No | / | 3.5 | / | / | -20.2 | / | / | / | 12.1 | / | / | / | / | |
| No | 3.9 | 3.0 | Yes | -16.8 | -15.4 | Yes | Yes | 14.2 | 14.8 | Yes | No | Yes | 16 | |
| No | 3.9 | 3.0 | Yes | -16.8 | -14.2 | Yes | Yes | 14.2 | 13.7 | No | Yes | No | 8 | |
| No | 3.9 | / | / | -16.8 | / | / | / | 14.2 | / | / | / | / | 2 | |
| No | / | 3.42 ± 0.05 | / | / | -20.13 ± 0.34 | / | / | / | 14.85 ± 0.63 | / | / | / | / | |
| / | 3.18 ± 0.02 | / | / | -18.27 ± 0.48 | / | / | / | 15.24 ± 0.55 | / | / | / | / | / | |
| No | 4.9 | 3.5 | Yes | -21.4 | -19.5 | Yes | Yes | 16.1 | 16.3 | Yes | No | Yes | 24.5 | |
| No | 3.4 | 3.4 | No | -18.9 | -19.1 | No | Yes | 15.5 | 15.9 | Yes | No | Yes | / | |
| No | 4.2 | 3.6 | Yes | -19.8 | -18.7 | Yes | Yes | 16.8 | 17.1 | Yes | No | Yes | / | |
| No | 3.4 | 3.4 | No | -19.1 | -19.1 | No | No | 16.4 | 16.8 | Yes | No | Yes | / | |
| No | 4.5 | 3.5 | Yes | -19.5 | -17.8 | Yes | Yes | 15.5 | 15.7 | Yes | No | Yes | 14.9 | |
| No | 3.6 | 3.3 | Yes | -18.2 | -17.9 | Yes | Yes | 14.9 | 15.1 | Yes | Yes | No | 7.9 | |
| No | 4.1 | 3.5 | Yes | -19.9 | -18.9 | Yes | Yes | 15.5 | 15.6 | Yes | No | Yes | 20.1 | |
| No | 3.5 | 3.4 | Yes | -18.8 | -18.9 | No | No | 15.1 | 15.3 | Yes | No | Yes | 7.9 | |
| No | 5.0 | 4.0 | Yes | -20.8 | -19.6 | Yes | Yes | 12.7 | 13.0 | Yes | No | Yes | 19.4 | |
| No | 3.5 | 3.3 | Yes | -19.4 | -19.2 | Yes | Yes | 12.3 | 12.7 | Yes | No | Yes | 17.1 | |
| No | 4.2 | 3.6 | Yes | -19.7 | -18.7 | Yes | Yes | 17.0 | 17.1 | Yes | No | Yes | 13.9 | |
| No | 3.5 | 3.4 | Yes | -18.7 | -18.4 | Yes | Yes | 16.4 | 16.7 | Yes | No | Yes | 9.3 | |
| No | 4.2 | 3.7 | Yes | -19.8 | -19.4 | Yes | Yes | 12.0 | 12.4 | Yes | No | Yes | / | |
| No | 4.0 | 3.5 | Yes | -19.5 | -18.8 | Yes | Yes | 16.8 | 17.0 | Yes | No | Yes | 11.7 | |
| No | 3.5 | 3.4 | Yes | -17.7 | -17.7 | No | No | 14.7 | 15.0 | Yes | No | Yes | 9.5 | |
| No | 4.1 | 3.6 | Yes | -18.2 | -17.3 | Yes | Yes | 15.0 | 15.1 | Yes | No | Yes | / | |
| / | / | / | / | / | -18.2 ± 0.14 | / | / | / | 16.55 ± 0.92 | / | / | / | / | |
| / | / | / | / | / | -18.9 ± 0.28 | / | / | / | 15.1 ± 0.14 | / | / | / | / | |
| / | / | 3.2 | / | / | -18.5 | / | / | / | 12.9 | / | / | / | / | |
| / | / | 3.9 | / | / | -19.3 | / | / | / | 13.0 | / | / | / | / | |
| / | / | 3.1 | / | / | -18.1 | / | / | / | 15.4 | / | / | / | / | |
| / | / | 3.3 | / | / | -18.4 | / | / | / | 16.4 | / | / | / | / | |
| / | / | 3.1 | / | / | -18.2 | / | / | / | 16.0 | / | / | / | / | |
| / | / | 3.4 | / | / | -18.4 | / | / | / | 17.0 | / | / | / | / | |
| / | / | 3.2 | / | / | -18.3 | / | / | / | 17.6 | / | / | / | / | |
| / | / | 3.4 | / | / | -18.2 | / | / | / | 18.4 | / | / | / | / | |

**Table S2.**

Table summarising the non-comprehensive literature review results in percent per category. The literature review included 40 research articles from 1997 to 2023.

| | All / Yes / Bligh & Dyer 1959 / Supernatant clear | None / No / Folch et al. 1957 / Several | Some / Other / One | Average / Not mentioned / Chloroform-Methanol |
|---|---|---|---|---|
| Number of Species | | | | 2.28 |
| Cetacean | 67.5 | 17.5 | 15.0 | |
| Mysticete | 20 | 70 | 10 | |
| Number of Tissues | | | | 1.88 |
| Skin | 32.5 | 32.5 | 35 | |
| Extraction Method | 22.5 | 5 | 52.5 | 20 |
| Solvent/s | | | 20 | 80 |
| Number of Extractions | 12.5 | 30 | 37.5 | 20 |
| Extraction Time | 55 | 45 | | |
| Test for Complete Extraction | 27.5 | 72.5 | | |
| C:N Before | 30 | 70 | | |
| C:N After | 57.5 | 42.5 | | |
